# Research on the Corrosion Detection of Rebar in Reinforced Concrete Based on SMFL Technology

**DOI:** 10.3390/ma17143421

**Published:** 2024-07-11

**Authors:** Hongsong Tian, Yujiang Kong, Bin Liu, Bin Ouyang, Zhenfeng He, Leng Liao

**Affiliations:** 1 Guizhou Bridge Construction Group Co., Ltd., Guiyang 550001, China; 2 State Key Laboratory of Mountain Bridge and Tunnel Engineering, Chongqing Jiaotong University, Chongqing 400074, China; 3 School of Civil Engineering, Chongqing Jiaotong University, Chongqing 400074, China

**Keywords:** rebar corrosion, reinforced concrete, SMFL technology, influence factors, prediction model, XGBoost

## Abstract

The corrosion damage of rebars is a leading cause of structural failure in reinforced concrete structures. Timely detection and evaluation of corrosion damage are crucial for ensuring structural safety. The self-magnetic flux leakage (SMFL) technology is often used due to its unique advantages in detecting corrosion damage of rebars. However, challenges persist in theoretically characterizing corrosion damage and exploring influencing factors. Therefore, the magnetic dipole theory model coupled with multiple-shaped defects is proposed and the influence of corrosion expansion force on the detection of corrosion damage is analyzed. The results show that the standard deviation of the magnetic field intensity induced by corrosion varied by up to 833%, while that induced by corrosion expansion force did not exceed 10%. So the changes in the SMFL field induced by corrosion damage play the dominant role and the influence of corrosion expansion force can be ignored. In addition, corrosion damage experiments on reinforced concrete based on the SMFL technology were conducted. The results indicate that the SFML curves of rebars change monotonically with the increasing corrosion degree. Significant variations in the curves correspond well with the locations of severe corrosion on the rebars. There is a positive relationship between the proposed magnetic parameters and the corrosion degree of the rebars. Furthermore, a corrosion damage evaluation model considering multiple parameters is developed to predict the corrosion degree of rebars. The prediction results demonstrate high accuracy, with an average absolute error of only 8.33%, which is within 10%.

## 1. Introduction

Reinforced concrete structures are widely utilized in building constructions, bridges, and various other fields due to their excellent mechanical properties. However, these structures are susceptible to several issues, including concrete carbonation, chloride erosion, and rebar corrosion, all of which are influenced by the service environment. These issues could significantly reduce the durability of reinforced concrete structures [1]. Among them, rebar corrosion caused by chloride erosion is the main factor considered in the degradation of structural durability. The corrosion diminishes the effective cross-sectional area of the steel bars, leading to degradation in the mechanical properties and a reduction in the bond between the reinforcement and the concrete. Moreover, the expansion pressure caused by corrosion products can result in corrosion-induced cracks in the protective concrete layer when it exceeds the tensile strength of the concrete [2,3,4]. Once the protective layer cracks, the ability of the protection layer to resist the invasion of harmful substances is weakened and new chloride transport channels are provided, thereby accelerating the deterioration in the durability of concrete structures [5,6]. Consequently, the detection of rebar corrosion damage in structures has garnered widespread attention.

Currently, methods for detecting corrosion damage in reinforced concrete structures can be categorized as destructive and non-destructive testing methods. Destructive testing methods require removing local concrete from the structure’s surface to directly assess the corrosion degree of the exposed rebars. However, due to the structural damage by destructive testing, non-destructive testing methods have become the primary approaches for detecting corrosion damage. The non-destructive testing methods for corrosion damage are based on changes in the material physical properties. These methods include electrochemical testing methods [7], acoustic testing methods [8], optical testing methods [9], and electromagnetic testing methods. In the field of corrosion damage detection, ultrasound and electromagnetic testing methods hold the dominant position. However, ultrasound testing methods make it difficult to quantitatively characterize the degree of rebar corrosion. Electromagnetic testing methods, including ground-penetrating radar [10], self-magnetic flux leakage (SMFL) [11,12,13], etc., have shown certain advantages in this regard. Among them, SMFL technology, which does not require external excitation compared to other methods, is particularly suitable for detecting the corrosion damage of rebars.

SMFL technology is a passive detection method based on changes in the magnetic properties to detect corrosion damage. Numerous studies have demonstrated the effectiveness of SMFL technology in detecting corrosion damage of rebars. For effective detection of corrosion damage, two critical elements must be considered: locating the corrosion and characterizing the degree of corrosion damage. The magnetic dipole model was initially proposed to identify the corrosion location. Over time, this model has evolved from the initial point magnetic dipole model to the current surface magnetic dipole model with in-depth theoretical research [14,15,16,17]. This development has enabled an effective description of the SMFL field in the corrosion area. Many experiments and studies on rebar corrosion damage have been conducted to verify the effectiveness of this model. The research results indicate that the intersection points and inflection points of the SMFL curves can be used to locate the corrosion position and identify the corrosion width [18,19,20,21]. This is consistent with the results obtained from the magnetic dipole theory model. This characteristic in the SMFL field has also been utilized to identify corrosion damage in prestressed steel strands [22].

Characterizing the degree of corrosion damage is also crucial for the safety assessment of structures. Therefore, many scholars have focused on quantitative assessment of corrosion damage based on the changes in the SMFL field. It has been found that there is a stable linear quantitative relationship between the change rate of the SMFL field and the corrosion degree of rebars [23]. Additionally, the Bayes algorithm was introduced to combine the magnetic parameters and the corrosion area parameters for quantitative assessment of corrosion, achieving an accuracy rate as high as 82.5% [24]. Furthermore, the factors influencing the results of the corrosion degree, such as the rebar diameter and protective layer thickness, were analyzed. The protective layer thickness affects the detection results by altering the lift-off height of the sensor [25]. Although the rebar diameter has certain effects on the changes in the SMFL field, the changes in the magnetic field induced by corrosion still play a dominant role [20]. Considering the influence of multiple factors, the discreteness of the SMFL field distribution during the corrosion process is relatively large. To address this, a grading assessment method was adopted and the support vector machine (SVM) statistical learning algorithm was introduced to evaluate its assessment system. Based on this, the SMFL technology was also innovatively applied to quantitative statistical evaluation of the bending strength and the characterization of the residual bearing capacity of corroded RC beams [26,27].

These studies have shown that the SMFL technology holds significant potential for the quantitative characterization of corrosion damage. The distribution characteristics of corrosion damage on rebars are multi-point distribution patterns. However, the current magnetic dipole models mostly focus on single defect distribution and there have been fewer studies on the magnetic dipole models with multiple coupled defects. In addition, the effect of corrosion expansion force on corrosion damage detection is not also explored. Therefore, this study conducted research on the magnetic dipole model coupled with multi-shaped defects and carried out corrosion damage detection experiments on reinforced concrete specimens. The effect of corrosion expansion force on corrosion damage detection is analyzed. Finally, this study achieved the prediction of corrosion damage in steel-reinforced concrete components based on a multi-parameter corrosion damage assessment model.

## 2. Materials and Methods

### 2.1. Materials Preparation

#### 2.1.1. Materials for Concrete Specimens

**Figure 1:**
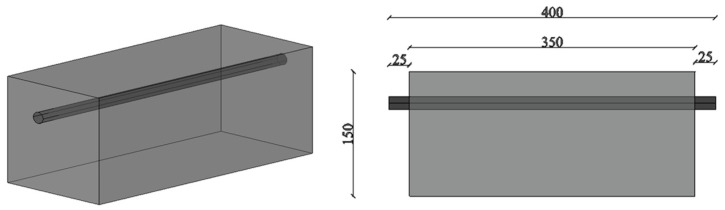
The dimensions of the specimen (unit: mm).

In the experiment, the reinforced concrete specimens were used as the main research objects. According to the experimental purpose and conditions, the specimen dimensions were designed as 150 mm × 150 mm × 350 mm, with a length of 400 mm for the main rebar, and 25 mm of each main rebar protruding from each end of the specimen, as shown in Figure 1. The design strength grade C30 of the concrete in code for the design of concrete structures (GB50010-2010) [28] was adopted. The concrete had a mix ratio of cement:fine aggregate:coarse aggregate:water of 461:512:1252:175 (kg/m^3^). The cement is 42.5 R grade ordinary Portland cement. Coarse aggregate is gravel with a diameter of 20 mm or less and fine aggregate is medium sand. The tensile strength *f*_tk_ of concrete is 2.01 MPa and the compressive strength *f*_ck_ of concrete is 20.1 MPa. The brand of rebars is HRB400. The chemical composition of the materials is shown in Table 1. The tensile strength *f*_y_ of the HRB400 rebar is 489 MPa.

**Figure 2:**
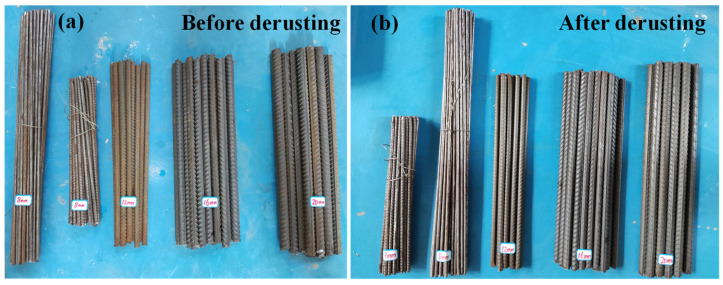
The appearance of the rebars before and after de-rusting.

**Figure 3:**
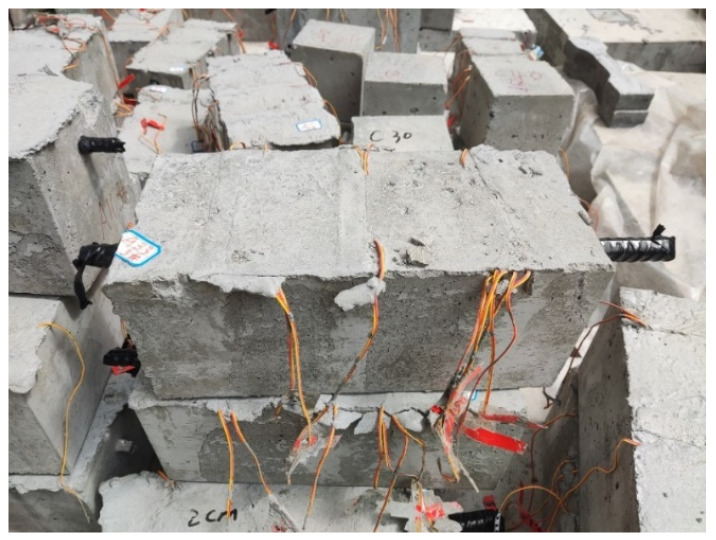
The reinforced concrete specimens after casting.

In addition, the effects of two variables on the SMFL field were considered in the experiment, namely the diameter of the rebars and the thickness of the concrete cover. For the rebar diameter (*D*), the specimens with three different rebar diameters of 12 mm, 16 mm, and 20 mm were designed. For the cover thickness (*T*), three different thicknesses of cover, 20 mm, 30 mm, and 40 mm, were designed. Before casting the test pieces, the surface of the rebars was de-rusted and the weight of the rebars was measured by using the balance, as shown in Figure 2. Each group consisted of three specimens to ensure the reliability and reproducibility of the experiment. The de-molded specimens are shown in Figure 3.

#### 2.1.2. Corrosive Materials for Specimens

The electrical-accelerated corrosion methods are often used in corrosion tests of reinforced concrete. The previous studies have shown differences between accelerated corrosion and natural corrosion. But the actual corrosion rate of rebars is essentially consistent with the theoretical corrosion rate when the rebar surface is passivated and fully in contact with the electrolyte [29]. The actual corrosion rate of the rebars can be accurately estimated according to Faraday’s law. So, the electrical-accelerated corrosion method is still used as the main method of corrosion when considering that the corrosion of rebars in natural environments is a relatively slow process. The electrical-accelerated corrosion methods require the preparation of an electrolyte solution, the DC power supply, and conductive carbon rods. The electrolyte solution is prepared using common salt, with a mass ratio of salt to water of 1:19, resulting in a 5% NaCl solution. The chosen DC power supply is an MS series switch-mode adjustable DC-regulated power supply with a rated power of 150 W. It has an output voltage range of 0–30 V (continuously adjustable) and an output current range of 0–5 A (continuously adjustable). The auxiliary carbon rods used are standard cylindrical carbon rods with a diameter of 10 mm.

### 2.2. The Methods for Testing

#### 2.2.1. The Corrosion Method of Specimens

**Figure 4:**
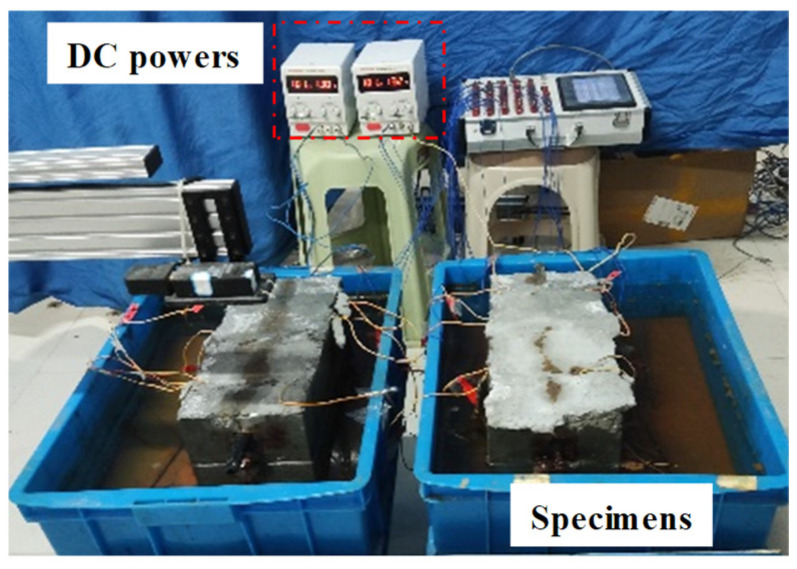
The layout of corroding specimens.

The corrosion of rebars for concrete components is often non-uniform. However, in earlier experiments, the corrosion of the rebars was mainly localized corrosion, which cannot adequately reflect the actual appearance of corrosion defects. Therefore, the semi-immersed electrical-accelerated method was used to simulate the overall corrosion condition of rebars inside concrete in the experiment. To ensure that there were corrosion defects at the designated locations without causing corrosion to the end rebars, epoxy resin was applied to the end sections of the specimens and the protruding end rebars were wrapped with waterproof tape. The specimens were immersed in a 5% NaCl solution for 48 h to facilitate the penetration of the salt solution to the surface of the rebar before the electrical-accelerated corrosion. During the experiment process, the electrical-accelerated corrosion experiment was conducted in the laboratory at room temperature (approximately 15 °C). The anode and cathode of the DC power supply were connected to the rebar in the concrete and the auxiliary carbon rod, which was placed in solution. Then, the lower half of the specimens were placed in the solution. Based on the electrolysis principle, the corrosion of the rebars was accelerated by controlling the current. So, the specimens were subjected to accelerated corrosion at a constant current of 0.2 A. After being powered on, ions driven by the DC power supply penetrate the concrete and reach the surface of the rebar, leading to the formation of a corrosion circuit. Under the long-term influence of the corrosion circuit, the degree of corrosion for rebars gradually intensifies. The layout of the experimental equipment is shown in Figure 4.

#### 2.2.2. The Collection Method of the SMFL Signal on Corroded Specimens

**Figure 5:**
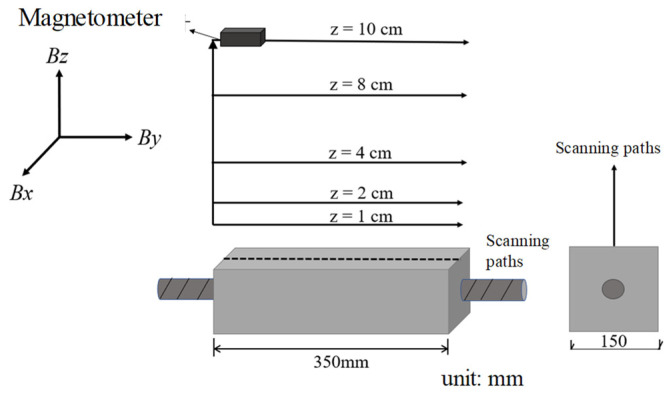
The scanning paths.

During the corrosion process of the specimens, the SMFL signals of the corroded specimens were collected by using the magnetometer HMR2300. The magnetometer can collect SMFL signals from three directions, with a range of ±2 Gs and an accuracy of 70 μGs. Since there is only one longitudinal rebar, the scanning paths were set directly above the longitudinal rebar, i.e., the centerline of the concrete specimen. The longitudinal length of the concrete specimen, which is 350 mm, was covered by the scanning paths. A total of 5 measurement heights were set with values of 1 cm, 2 cm, 4 cm, 8 cm, and 10 cm above the surface of the specimen. The scanning paths are illustrated in Figure 5. In order to obtain as much SMFL signal data as possible, the SMFL signals were collected along the predetermined scanning paths every hour.

### 2.3. Theory Background of Testing

**Figure 6:**
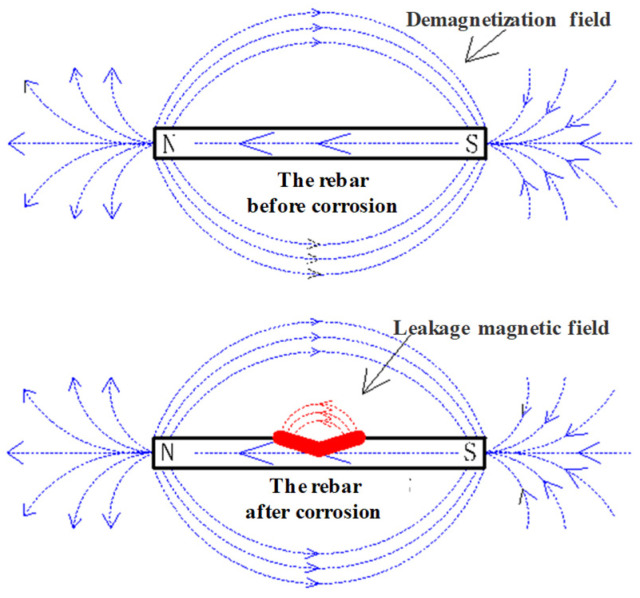
The magnetic field of rebars before and after corrosion.

Under the long-term influence of geomagnetic fields, a weak magnetic field will gradually appear on the surface of rebars. When rebars are subjected to corrosion, their cross-sectional area inevitably decreases, resulting in obvious concave shapes in areas damaged by corrosion. The permeability of this area changes from the high permeability of iron to the low permeability of air. Consequently, a leakage magnetic field opposite the direction of the rebars’ magnetic field forms at the corrosion position, as shown in Figure 6. Because the generated rust exists inside the structure of the reinforced concrete, the corroded rebar bears an additional corrosion expansion force. Therefore, the measured magnetic field of rebars is actually composed of multiple magnetic components, including the geomagnetic field, the magnetic field induced by the geomagnetic field, the SMFL field induced by corrosion defects, and the magnetic field induced by corrosion expansion force. The specific expression is as follows:

(1) $$H=H_{e}+H_{r}+H_{c}+H_{\sigma }$$

where *H_e_* is the geomagnetic field, *H_r_* is the magnetic field induced by the geomagnetic field, *H_c_* is the SMFL field induced by corrosion damage, and *H_σ_* is the magnetic field induced by corrosion expansion force. Compared with the magnetic field of the rebar before corrosion, the change in the magnetic field after corrosion is due to the presence of the SMFL field induced by corrosion damage and the magnetic field induced by corrosion expansion force. Therefore, analyzing the expression of the magnetic field induced by corrosion damage and corrosion expansion force is helpful in obtaining the magnetic field distribution of reinforced concrete structures after corrosion.

#### 2.3.1. The Magnetic Field Induced by Corrosion

**Figure 7:**
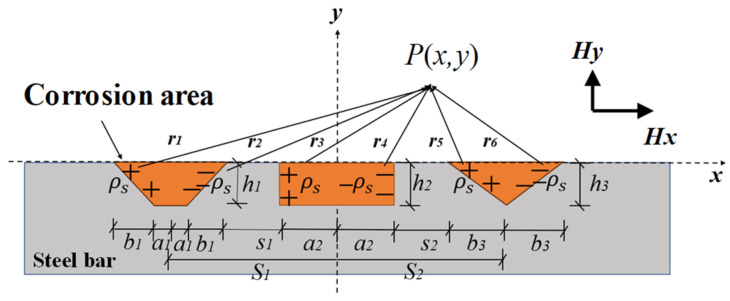
The magnetic dipole model for multiple shaped defects.

In practical engineering, the corrosion of rebars inside reinforced concrete members is often dominated by patchy corrosion [30]. This type of corrosion is characterized by its distribution over a wide area of the rebar surface, shallow corrosion depth, random distribution of corrosion locations, and varying degrees of corrosion. Therefore, it is necessary to explore a magnetic dipole model that considers multiple coupled defects. In addition to the randomness of the positions and the number of corrosion defects, the complex shapes of the defects directly affect the SMFL field on the material surface. Therefore, a two-dimensional magnetic dipole model is adopted to study the behavior of the SMFL field of multiple corrosion defects. The magnetic dipole model for multiple-shaped defects is illustrated in Figure 7. In the figure, *ρ_s_* is the magnetic charge density, *h* represents the depth of the curve, *a* represents the width of the bottom edge of the defect, *b* is the horizontal width of the diagonal edge of the defect, and *S* is the horizontal spacing between defects.

The magnetic field at any point *P*(*x*, *y*) in space by the surface magnetic charge with depth *η*, width *dη*, and magnetic charge density *ρ_s_* is given by [31]

(2) $$\begin{array}{l} d{\vec{H}}_{1}=\left[\begin{array}{l} dH_{1x}\\ dH_{1y} \end{array}\right]=\frac{\rho_{s}ds}{2\pi \mu_{0}r_{1}^{2}}{\vec{r}}_{1}\\ =\frac{\rho_{s}\sqrt{1+b_{1}^{2}/h_{1}^{2}}d\eta }{2\pi \mu_{0}\left[{\left(x+\eta b_{1}/h_{1}+a_{2}+s_{1}+2a_{1}+2b_{1}\right)}^{2}+{\left(y-\eta \right)}^{2}\right]}\cdot \left(\begin{array}{l} x+\eta b_{1}/h_{1}+a_{2}+s_{1}+2a_{1}+2b_{1}\\ y-\eta \end{array}\right) \end{array}$$

(3) $$\begin{array}{l} d{\vec{H}}_{2}=\left[\begin{array}{l} dH_{2x}\\ dH_{2y} \end{array}\right]=-\frac{\rho_{s}ds}{2\pi \mu_{0}r_{2}^{2}}{\vec{r}}_{2}\\ =-\frac{\rho_{s}\sqrt{1+b_{1}^{2}/h_{1}^{2}}d\eta }{2\pi \mu_{0}\left[{\left(x+a_{2}+s_{1}-\eta b_{1}/h_{1}\right)}^{2}+{\left(y-\eta \right)}^{2}\right]}\cdot \left(\begin{array}{l} x+a_{2}+s_{1}-\eta b_{1}/h_{1}\\ y-\eta \end{array}\right) \end{array}$$

(4) $$d{\vec{H}}_{3}=\left[\begin{array}{l} dH_{3x}\\ dH_{3y} \end{array}\right]=\frac{\rho_{s}ds}{2\pi \mu_{0}r_{3}^{2}}{\vec{r}}_{3}=\frac{\rho_{s}d\eta }{2\pi \mu_{0}\left[{\left(x+a_{2}\right)}^{2}+{\left(y-\eta \right)}^{2}\right]}\left(\begin{array}{l} x+a_{2}\\ y-\eta \end{array}\right)$$

(5) $$d{\vec{H}}_{4}=\left[\begin{array}{l} dH_{4x}\\ dH_{4y} \end{array}\right]=-\frac{\rho_{s}ds}{2\pi \mu_{0}r_{4}^{2}}{\vec{r}}_{4}=-\frac{\rho_{s}d\eta }{2\pi \mu_{0}\left[{\left(x-a_{2}\right)}^{2}+{\left(y-\eta \right)}^{2}\right]}\left(\begin{array}{l} x-a_{2}\\ y-\eta \end{array}\right)$$

(6) $$\begin{array}{l} d{\vec{H}}_{5}=\left[\begin{array}{l} dH_{5x}\\ dH_{5y} \end{array}\right]=\frac{\rho_{s}ds}{2\pi \mu_{0}r_{5}^{2}}{\vec{r}}_{5}\\ =\frac{\rho_{s}\sqrt{1+b_{3}^{2}/h_{3}^{2}}d\eta }{2\pi \mu_{0}\left[{\left(x-a_{2}-s_{2}+\eta b_{3}/h_{3}\right)}^{2}+{\left(y-\eta \right)}^{2}\right]}\cdot \left(\begin{array}{l} x-a_{2}-s_{2}+\eta b_{3}/h_{3}\\ y-\eta \end{array}\right) \end{array}$$

(7) $$\begin{array}{l} d{\vec{H}}_{6}=\left[\begin{array}{l} dH_{6x}\\ dH_{6y} \end{array}\right]=-\frac{\rho_{s}ds}{2\pi \mu_{0}r_{6}^{2}}{\vec{r}}_{6}\\ =-\frac{\rho_{s}\sqrt{1+b_{3}^{2}/h_{3}^{2}}d\eta }{2\pi \mu_{0}\left[{\left(x-a_{2}-s_{2}-2b_{3}-\eta b_{3}/h_{3}\right)}^{2}+{\left(y-\eta \right)}^{2}\right]}\cdot \left(\begin{array}{l} x-a_{2}-s_{2}-2b_{3}-\eta b_{3}/h_{3}\\ y-\eta \end{array}\right) \end{array}$$

where *µ*_0_ is the vacuum magnetic permeability, *r* is the distance between the magnetic charge and point *P*, and the subscripts 1 to 6 refer to the different defective surfaces from left to right. The subscripts 1 refers to the leftmost defect surface and the subscripts 6 refers to the rightmost defect surface. Magnetic components (*H_x_* and *H_y_*) can be obtained according to Equation (8).

(8) $$\begin{array}{l} H_{x(y)}=\int_{-h_{1}}^{0}dH_{1x(y)}+\int_{-h_{1}}^{0}dH_{2x(y)}+\int_{-h_{2}}^{0}dH_{3x(y)}\\ +\int_{-h_{2}}^{0}dH_{4x(y)}+\int_{-h_{3}}^{0}dH_{5x(y)}+\int_{-h_{3}}^{0}dH_{6x(y)} \end{array}$$

#### 2.3.2. The Magnetic Field Induced by Corrosion Expansion Force

The volume expansion of rebars caused by corrosion products after corrosion results in tensile stress on the concrete cover layer. Before cracking, the concrete cover layer will transmit radial compressive stress to the rebars’ corrosion products. Under the influence of the geomagnetic field and corrosion expansion force, the magnetic field of the rebars changes accordingly. For the ferromagnetic materials, the stress–magnetization effect is a complex phenomenon. Assuming a constant external magnetic field and a constant temperature environment, the magnetization state of ferromagnetic materials in the equilibrium state of the magnetostriction effect can be expressed as

(9) $$M=M_{s}\left[\coth\left(\frac{H_{total}}{a}\right)-\frac{a}{H_{total}}\right]$$

where *M* is the non-hysteretic magnetization, *M_s_* is the saturation magnetization of the material, *H_total_* is the equivalent field intensity, and *a* is the magnetization model parameter (A/m). *H_total_*, the equivalent field intensity experienced by the material under the action of an external magnetic field and external loads, can be expressed as [32]

(10) $$H_{total}=H_{0}+\alpha M+H_{\sigma }^{e}$$

In the equation, *α* denotes the material parameter, reflecting the interaction between magnetic domains; *H_σ_^e^* denotes the equivalent field induced by the force–magnetic coupling effect, which is related to the magnetostriction of the material. It can be expressed as

(11) $$\lambda (\sigma ,M)=\left(1-\frac{\beta \sigma }{\sigma_{s}}\right)\frac{\lambda_{s}M^{2}}{M_{s}^{2}}$$

where *σ* is the stress and *β* is the parameter controlling the stress–magnetization effect. *λ_s_* is the material’s saturation magnetostriction coefficient. For isotropic ferromagnetic materials, the magnetostatic energy density function caused by the magnetoelastic effect can be expressed as

(12) $$A_{\sigma }^{e}=\frac{3}{2}\sigma \lambda$$

The magnetoelastic equivalent field can be expressed as the derivative of the magnetoelastic energy density function with respect to the magnetization intensity and its calculation result is as follows:

(13) $$H_{\sigma }^{e}=\frac{1}{\mu_{0}}\frac{dA_{\sigma }^{e}}{dM}=\frac{3\sigma }{\mu_{0}}\left(1-\frac{\beta \sigma }{\sigma_{s}}\right)\frac{\lambda_{s}M}{M_{s}^{2}}$$

So, the equivalent field intensity can be finally expressed as

(14) $$H_{total}=H_{0}+\alpha M+\frac{3\sigma }{\mu_{0}}\left(1-\frac{\beta \sigma }{\sigma_{s}}\right)\frac{\lambda_{s}M}{M_{s}^{2}}$$

In the weak magnetization state, if the Langevin function coth(x) in Equation (9) is replaced by the first-order approximation of its Taylor expansion, it can be simplified to

(15) $$M=M_{s}H_{total}/(3a)$$

Substituting Equation (14) into Equation (15) leads to

(16) $$\frac{3aM}{M_{s}}=H_{0}+\alpha M+\frac{3\sigma }{\mu_{0}}\left(1-\frac{\beta \sigma }{\sigma_{s}}\right)\frac{\lambda_{s}M}{M_{s}^{2}}$$

Combining the above equations, the analytical expression for magnetization with respect to stress and external magnetic field can be obtained in the weak magnetization state.

(17) $$M\left(\sigma ,H_{0}\right)=\frac{H_{0}M_{s}^{2}}{3aM_{s}-\alpha M_{s}^{2}-3\sigma \left(1-\beta \sigma /\sigma_{s}\right)\lambda_{s}/\mu_{0}}$$

Therefore, the magnetization intensity induced by the geomagnetic field and corrosion expansion force can be obtained. By combining Equations (1), (8) and (17), the magnetic field strength of corroded reinforced concrete structures can be calculated theoretically.

#### 2.3.3. The Magnetic Field Induced by the Coupling Effect of Corrosion and Corrosion Expansion Force

Based on the magneton theory, the magnetic charge density can be obtained as

(18) $$\rho_{s}=\mu_{0}M\left(\sigma ,H_{0}\right)$$

**Figure 8:**
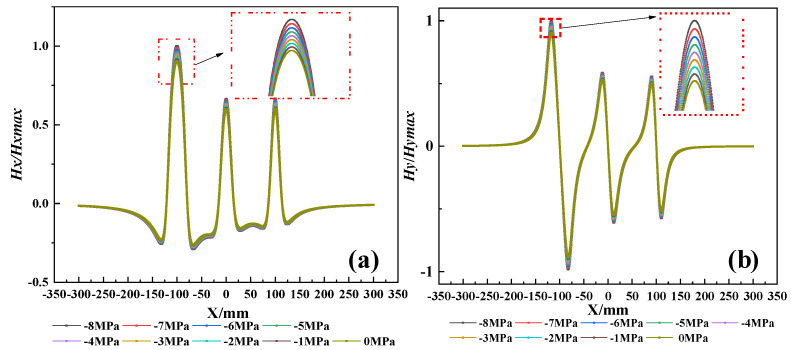
The SMFL curves with stress, (**a**) The *Hx* curves with stress. (**b**) The *Hy* curves with stress.

**Figure 9:**
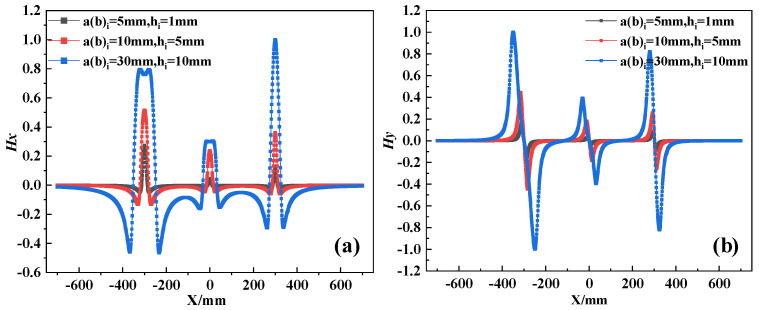
The SMFL curves with corrosion degree, (**a**) The *Hx* curves with corrosion degree. (**b**) The *Hy* curves with corrosion degree.

The magnetic field intensity *Hx* and *Hy* at any position in space can be obtained by combining Equations (8), (17) and (18). To investigate the effects of corrosion expansion force and corrosion degree on magnetization intensity, with defect parameters *a_i_* = 5, 10, 30 mm, *b_i_* = 10 mm, *s_i_* = 100 mm, *h_i_* = 10 mm, and *z* = 10 mm. Based on the study conducted by Jin et al. [33] it was found that when the thickness of the protective layer (c) relative to the diameter of the steel bar attains a ratio of 2.5, the structure experiences a maximum corrosion expansion force of 3.62 MPa under uniform corrosion conditions. Conversely, under non-uniform corrosion, the maximum rust expansion force escalates to 8.57 MPa. So, the corrosion expansion stress is set from −8 MPa to 0 MPa and the external magnetic field is set to 40 A/m. For ease of analyzing the effect of corrosion expansion force, the calculation results are normalized to the peak value, as shown in Figure 8 and Figure 9.

**Figure 10:**
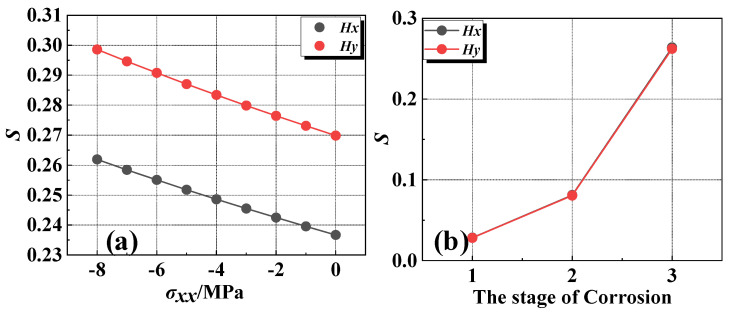
The standard deviation with the corrosion expansion force and corrosion degree. (**a**) The standard deviation with the corrosion expansion force. (**b**) The standard deviation with the corrosion degree.

From the results, it is evident that the curves of the *H_x_* component exhibit peaks at the defect position, while the curves of the *H_y_* component show positive and negative peaks symmetrically around the center of the defect. Additionally, the magnetic field intensity monotonically increases with the increase in stress and the corrosion degree. However, the change in magnetic field intensity caused by corrosion expansion force is much smaller compared to that caused by corrosion. To quantify the effects of both factors, the standard deviation of the magnetic field intensity variation is extracted, as shown in Figure 10. At the same level of corrosion, the standard deviation change caused by 5 MPa is 6.3%. In contrast, the standard deviation variation from the first stage of corrosion to the second stage is 186%. And from the first stage to the third stage, it is 833%. Therefore, corrosion expansion stress does not significantly affect the qualitative diagnosis of rebar corrosion. When multiple corrosion defects are presented, the overall corrosion diagnosis of the rebars cannot be judged solely by the numerical values of individual peaks and troughs of the waveform. Therefore, it requires a comprehensive judgment based on the overall dispersion of the magnetic curves.

## 3. Results and Discussion

### 3.1. Results of Corrosion

**Figure 11:**
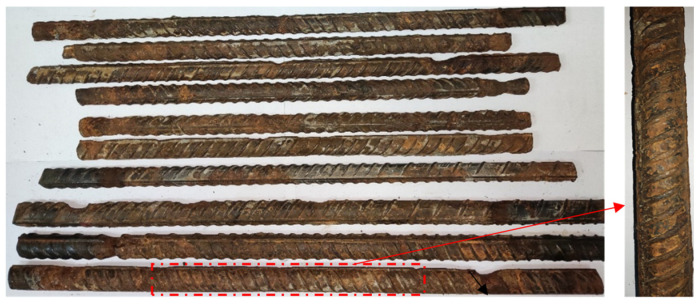
The corrosion condition of the rebars.

At the end of the accelerated corrosion test, the reinforced concrete specimens were broken to obtain the corroded rebars. Firstly, the concrete covering the rebars was removed. Then, the rebars were immersed in a rust remover solution and subsequently neutralized in aqueous Ca(OH)_2_ solution. After the rebars naturally dried, the appearance of the corroded rebars was obtained, as shown in Figure 11. Most corrosion defects were mainly pitting corrosion. Finally, the rebars were weighed to obtain their weight *m_a_* after corrosion.

The theoretical corrosion mass of the rebars can be calculated according to Faraday’s first law, as shown in the following equation [34]:

(19) $$m_{t}=\frac{I\times T}{F}\times \frac{M}{n}$$

where the mass lost due to corrosion of metal is represented by *m_t_*. *I* is the average electric current intensity. *T* represents the time of electrified corrosion. *F* represents the Faraday constant whose value is 96,484 (C/mol). *M* represents the molar mass of iron, *M* = 55.487. *n* is the number of electrons consumed when an iron atom is converted to Fe^2+^, *n* = 2.

*C* is defined as the corrosion rate of internal rebars. It can be calculated based on the corrosion mass of the rebars *m_t_*.

(20) $$C=\frac{m_{t}}{m_{0}}$$

where *m*_0_ is the initial mass of the rebars in the specimen. $m_{0}=\pi r^{2}\rho l$. *ρ* is the density of iron. *l* is the length of the longitudinal rebar. The theoretical corrosion mass and average corrosion rate of each specimen can be calculated using Equations (19) and (20). The actual corrosion *m_t_* is obtained by subtracting the mass *m_a_* after corrosion from the initial mass *m_0_* of the rebars. *m_t_* and *C* are two primary indicators that are used to describe the degree of corrosion.

The actual corrosion rates and theoretical corrosion rates calculated using Equation (20) are shown in Table 2. From Table 2, it can be observed that there is some difference between the actual corrosion rate and the theoretical corrosion rate. The main reason for the difference is the inadequate anti-corrosion measures at the ends of the specimens, resulting in corrosion of the rebars at the end sections. Additionally, differences in the passivation film of the rebars also contribute to the discrepancy between the actual and theoretical corrosion rates, leading to the actual corrosion rate being smaller than the theoretical corrosion rate.

### 3.2. Analysis of SMFL Signals during the Corrosion Process of Specimens

**Figure 12:**
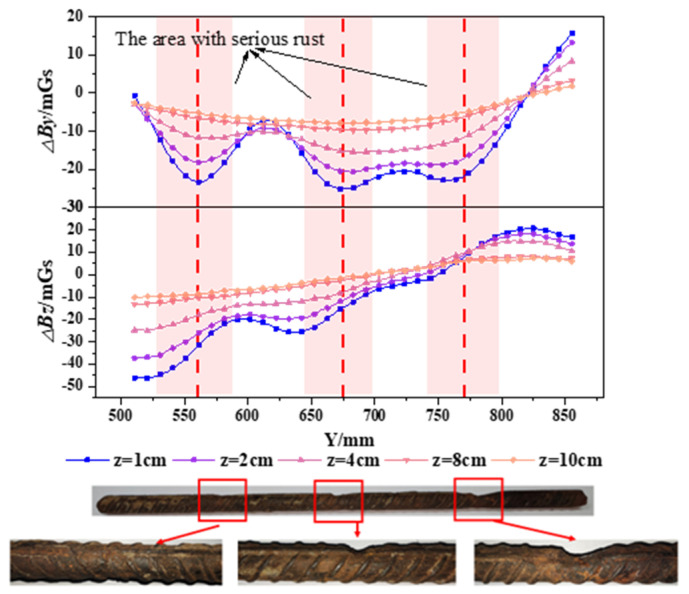
Δ*By* and Δ*Bz* curves of the A32-1 specimen after corrosion.

**Figure 13:**
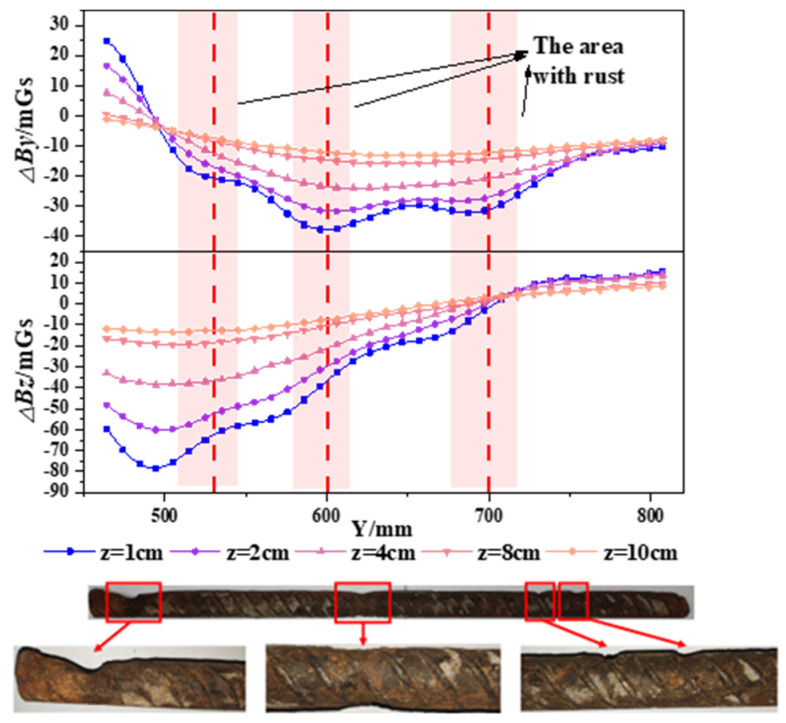
Δ*By* and Δ*Bz* curves of the B32-3 specimen after corrosion.

To better analyze the changes in SMFL signals caused by corrosion, the SMFL signals of the corroded specimens were subtracted from their initial SMFL signals. The results are denoted as △*B_y_* (tangential magnetic component) and △*B_z_* (normal magnetic component). Since the trends of change for each specimen are similar, the A32-1 specimen and B32-3 specimen are selected as examples for analysis. The results are shown in Figure 12 and Figure 13. Figure 12 illustrates the changes in △*B_y_* and △*B_z_* curves for the A32-1 specimen at different measurement heights, while Figure 13 shows the changes in △*B_y_* and △*B_z_* curves for the B32-3 specimen at different measurement heights. From the figures, it can be observed that the change in amplitude in both △*B_y_* and △*B_z_* curves gradually decreases with increasing measurement height. However, the △*B_y_* and △*B_z_* curves exhibit different distribution patterns after corrosion. There are multiple troughs for the △*B_y_* curves, while the △*B_z_* curves exhibit intersection points. Through analysis, this change in distribution pattern is attributed to the presence of corrosion damage. The troughs in the △*B_y_* curves and the intersection point in the △*B_z_* curves correspond to the severely corroded areas of the rebars. This distribution trend is consistent with the theoretical analysis results. To verify the accuracy of the results, the severely corroded areas from the △*B_y_* and △*B_z_* curves are compared to the actual corrosion. It is found that the results obtained are consistent with the actual corrosion locations. Additionally, only one severely corroded area can be identified from the △*B_z_* curves, which have only one intersection point. Therefore, compared to the △*B_z_* curves, the △*B_y_* curves can more accurately identify the severely corroded areas of the rebars.

**Figure 14:**
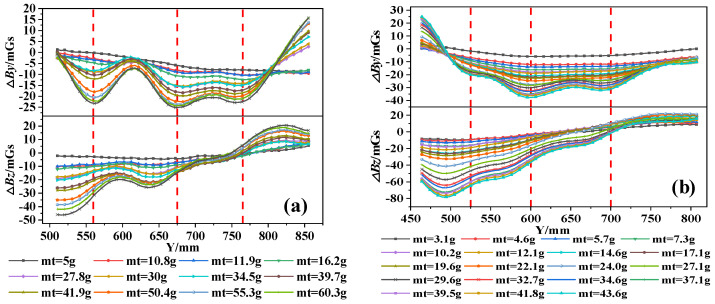
The Δ*By* and Δ*Bz* curves with different corrosion masses: (**a**) A32-1 specimen and (**b**) B32-3 specimen.

**Figure 15:**
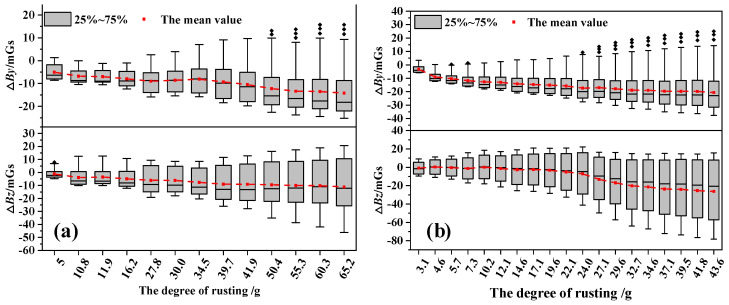
The box plots of Δ*By* and Δ*Bz* with different corrosion masses: (**a**) A32-1 specimen and (**b**) B32-3 specimen.

Figure 14 shows the △*B_y_* and △*B_z_* curves of the A32-1 specimen and B32-3 specimen at a measurement height of 1 cm. It can be observed that the curve characteristics of the specimens are similar. In the case of mild corrosion, the distribution pattern of the △*B_y_* and △*B_z_* curve remains roughly unchanged, reflecting the weak impact of corrosion. However, the amplitude of the curves becomes significant with the increase in corrosion mass. Specifically, the amplitude and width of the peaks and troughs in the △*B_y_* curves experience an increase: concurrently, the slope of the △*B_z_* curves becomes steeper and the disparity between the positive and negative peaks widens. This suggests a direct and substantial positive relationship between the escalation of corrosion and the alteration in the intensity of the magnetic field signal. To further analyze the dispersion of the SMFL curves and the variation in SMFL intensity with corrosion severity, the box plots of the SMFL curves are obtained, as shown in Figure 15. The results show that the distribution range of △*B_y_* and △*B_z_* increases gradually, as does the length of the box with increasing corrosion mass, which represents the middle 50% of the data. Additionally, the mean absolute value also increases with increasing corrosion mass. Overall, the change in the overall dispersion of the curves can be used as a basis for constructing quantitative indicators of the corrosion severity of rebars inside concrete specimens.

### 3.3. Corrosion Degree Assessment of the Rebar Based on XGBoost Algorithm

#### 3.3.1. The Magnetic Parameters of the Characterizing Corrosion Degree

Based on the above analysis, the gradient values can reflect the degree of corrosion damage to some extent. The gradient values along a single scanning path reflect the damage level of the entire specimen. Therefore, the integration is introduced from the mathematical system in order to reflect the corrosion damage of the specimens under a certain corrosion state. Integration can achieve the cumulative effect of a variable, which is exactly in line with the need of this experiment. It is to accumulate the damage level of each measurement position along the scanning path to represent the damage level of the component under this corrosion state. Since the gradient values at each measurement point along each scanning path are discrete, the absolute values of each gradient value are summed to achieve the effect of integration along the scanning path. The parameters *K_y_* and *K_z_,* which represent the integral of the absolute gradient values of the SMFL signal along the scanning path, are defined, as shown in Equation (21).

(21) $$K_{y}=\sum \left|d\Delta B_{y}/dx\right|,K_{z}=\sum \left|d\Delta B_{z}/dx\right|$$

**Figure 16:**
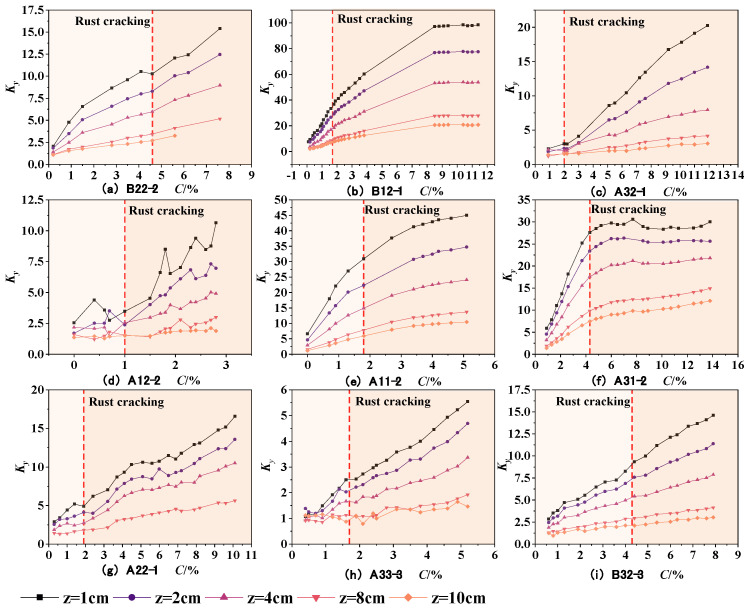
The changes in *K_y_* with the corrosion rate.

**Figure 17:**
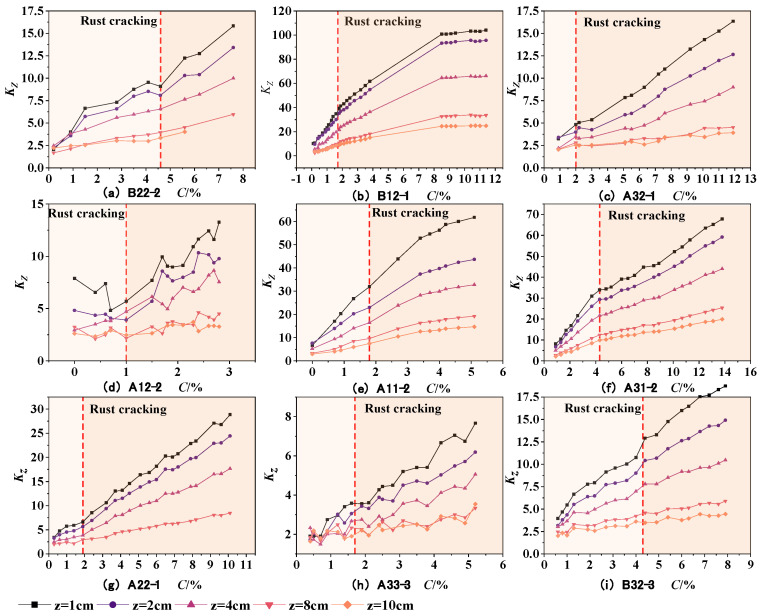
The changes in *K_z_* with the corrosion rate.

In the equation, △*B_y_* and △*B_z_* represent the tangential and normal SMFL signals, respectively. The results obtained are shown in Figure 16 and Figure 17. As can be seen from the figures, the *K_y_* and *K_z_* of each specimen exhibit similar variations. With an increase in the corrosion rate, the magnetic parameters *K_y_* and *K_z_* show an approximately linear increase, indicating a good positive correlation with the corrosion rate. As the intensity of the measurement height intensifies, the slope of the curves depicted in the figures progressively diminishes, approaching a more horizontal orientation. This also implies that a reduced measurement height enhances the precision in detecting corrosion damage. Furthermore, the magnetic parameters *K_y_* and *K_z_* exhibit similar slopes before and after rust expansion and cracking. This suggests that these magnetic parameters can effectively represent the overall corrosion rate of the rebars before and after rust expansion and cracking. On the other hand, it also suggests that the corrosion expansion stress generated by the corrosion products has a minimal effect on the magnetic parameters *K_y_* and *K_z_* in characterizing the overall corrosion rate of the rebars. From the above, it can be concluded that the magnetic parameters *K_y_* and *K_z_* can quantitatively characterize the corrosion degree of rebars inside concrete under overall corrosion conditions.

**Figure 18:**
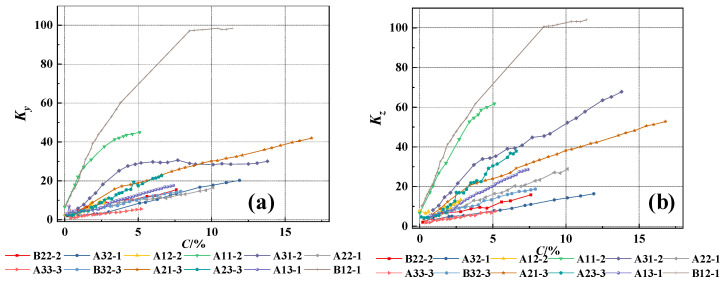
The changes in *K_y_* and *K_z_* with the corrosion rate when z = 1 cm, (**a**) The changes in *K_y_*; (**b**) The changes in *K_z_*.

The preceding analysis has shown that the measurement height has a significant impact on the SMFL signal. In order to ensure a high level of accuracy when analyzing the correlation between magnetic indicators and corrosion rate, only the SMFL quantification indicators with a measurement height of 1cm will be analyzed in the subsequent analysis. Figure 18 shows the relationship between the magnetic parameters *K_y_* and *K_z_* and the corrosion rate for representative specimens from different groups when the measurement height is 1cm. From the figures, it can be observed that the slope of curves for two groups of specimens is larger compared to the other groups. Apart from differences in diameter and protective layer thickness, the main reason for this is that the magnitude of SMFL signal change at adjacent positions is large when the corrosion of the steel bars is concentrated locally. To weaken the influence, the initialization correction is applied, as shown in the following equation

(22) $$K_{y}=\frac{K_{yi}}{K_{y0}},K_{z}=\frac{K_{zi}}{K_{z0}}$$

**Figure 19:**
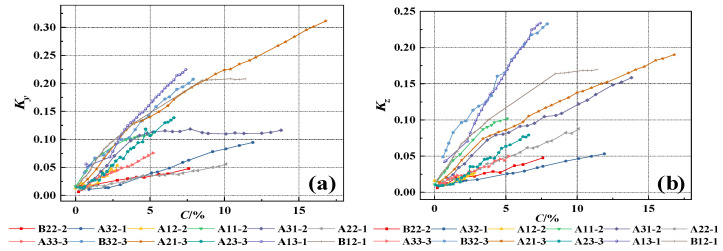
The corrected changes in *K_y_* and *K_z_* with the corrosion rate when z = 1 cm, (**a**) The corrected changes in *K_y_*; (**b**) The corrected changes in *K_z_*.

In the equation, *K_yi_* and *K_zi_* represent the magnetic parameters obtained at the *i_th_* corrosion rate, while *K_y_*_0_ and *K_z_*_0_ represent the initial magnetic parameters. The results obtained are shown in Figure 19. Compared to Figure 18, the dispersion of the magnetic parameters of specimens from different groups is reduced after the correction but there is still some dispersion. The dispersion of *K_y_* and *K_z_* stems from factors such as the dynamic magnetization intensity *M* during the experiment, differences in the protective layer thickness, and the diameter of rebars. This dispersion phenomenon is reasonable and also the reason for using machine learning algorithms in the subsequent analysis.

#### 3.3.2. Assessment of the Rebar Corrosion Degree Based on Multiple Parameters

XGBoost is an ensemble learning algorithm based on decision trees. The basic components of XGBoost are decision trees and there is a sequential relationship between these decision trees [35]. Specifically, the prediction results of the previous tree are comprehensively considered to more effectively adjust the distribution of training samples in the construction process of subsequent decision trees. This process aims to correct any biases that may exist in the previous decision tree so that the training samples that were previously misclassified can receive more attention and weight in subsequent training. Therefore, each decision tree can more accurately capture the complex relationships in the data based on the adjusted sample distribution, further improving the overall performance of the model. In addition, the generation of each decision tree is based on the entire dataset. Therefore, the generation of each decision tree can be viewed as an independent and complete process. This generation method based on the entire dataset helps each decision tree to fully utilize the information in the data and play the greatest role in the model.

In the computational process of XGBoost, the objective function can be represented as Equation (23) for a model with *t* iterations. Here, $\overset{\wedge (t-1)}{y}$ represents the prediction result of the model after *t* − *1* rounds of iterations and $f_{t}(x_{i})$ represents a new function introduced in the current iteration. In addition, *C* represents the constant term in the objective function, while $\Omega (f_{t})$ represents the regularization term of the regression tree, whose specific expression is shown in Equation (24). In this regularization term, *γ* and *λ* are hyperparameters used to control the number of leaf nodes and the weights of the leaf nodes of the regression tree, respectively. *T* represents the total number of leaf nodes of the regression tree and *ω_j_* represents the weight of each leaf node [35].

(23) $$L^{t}=\sum_{i=1}^{n}L\left[\left(y_{i},{\overset{\wedge }{y}}^{\left(t-1\right)})+f_{t}(x_{i}\right)\right]+\Omega (f_{t})+C$$

(24) $$\Omega (f_{t})=\gamma T+\frac{1}{2}\lambda \sum_{j=1}^{T}\omega_{j}^{2}$$

During the gradient descent process in XGBoost, it is not necessary to specify the loss function. Instead, optimization for leaf splitting is performed relying on the input data. In XGBoost, each leaf node contains a certain number of samples. *G_j_* and *H_j_* are defined in Equation (24) and Equation (25), respectively, representing the sum of the first- and second-order derivatives of the samples contained in leaf node *j*. With the decision tree structure fixed, the weight *ω_j_* of each leaf node *j* can be solved, as shown in Equation (26). Then, the Taylor expansion of the objective function is performed to obtain the optimal objective value of the objective function [35].

(25) $$G_{j}=\sum_{i\in I_{j}}g_{j}$$

(26) $$H_{j}=\sum_{i\in I_{j}}h_{i}$$

(27) $$\omega_{j}=-\frac{G_{j}}{H_{j}+\lambda }$$

(28) $$L^{t+1}=-\frac{1}{2}\sum_{j=1}^{T}\frac{G_{j}^{2}}{H_{j}+\lambda }+\gamma T$$

Before training the model, it is essential to determine the hyperparameters. Appropriate hyperparameters can optimize and enhance the model’s performance. The hyperparameters of XGBoost include the maximum tree depth, learning rate, number of base learners, etc. To determine the best combination of all hyperparameter values, a grid search method with an adaptive optimization search capability [36] is used to traverse all combinations of candidate hyperparameters. Five-fold cross-validation is used to evaluate the performance of each candidate parameter combination. In this paper, accuracy is used as the metric to measure the performance of each hyperparameter combination. Since it is not feasible to exhaust all possible values, a subset of candidate values is selected for each hyperparameter and the final selection of the model’s hyperparameters is shown in Table 3.

Based on the above analysis, it is observed that the magnetic parameters of specimens from different diameters and protective layer thickness exhibit variability. Therefore, two magnetic parameters, along with the diameter of the rebars and the thickness of the protective layer, are chosen as feature inputs. All 385 sets of experimental samples are used as the model sample data. Among them, 80% are randomly selected as the training set and the remaining 20% are used as the test set for predicting the corrosion rate, *C,* of the rebars. An XGBoost model is used to analyze the corrosion rate *C* of the training set and its corresponding feature values. The corresponding prediction model is established. The trained prediction model is then used to predict the corrosion rate *C* for the test set.

**Figure 20:**
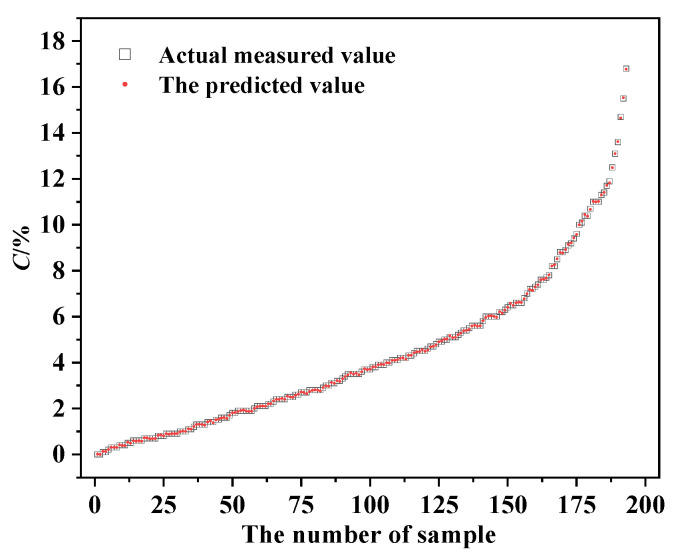
The predicted results of the training set samples.

Using the established XGBoost model, regression tests are conducted on 308 training samples. Here, the results of 40 training samples are listed in Table 4 and shown in Figure 20. From Table 4, it can be seen that the model exhibits good regression performance on the training set samples. The goodness of fit (R^2^) on the training set is 0.99, the standard deviation (SD) is 0.016, and the root mean square error (RMSE) is 0.025, demonstrating the feasibility of this method.

**Figure 21:**
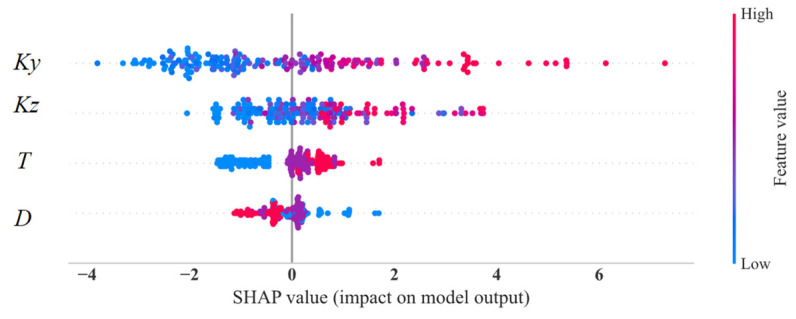
SHAP summary plot.

**Figure 22:**
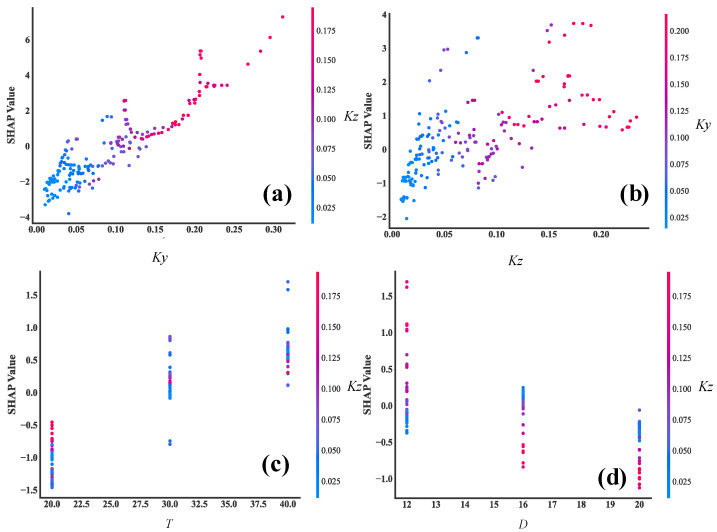
SHAP dependence plot. (**a**) SHAP dependence plot of *Ky*, (**b**) SHAP dependence plot of *Kz*, (**c**) SHAP dependence plot of *T*, (**d**) SHAP dependence plot of *D*.

**Figure 23:**
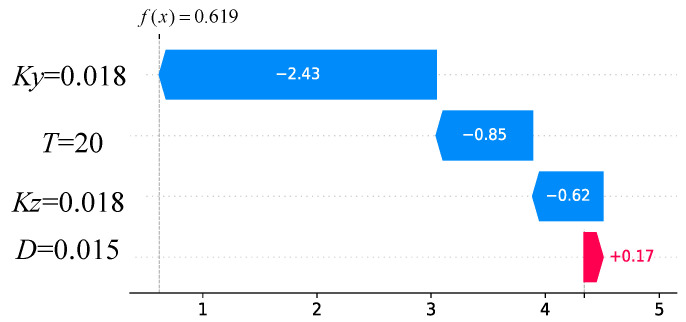
SHAP waterfall plot.

In order to evaluate the importance and sensitivity of each input feature, the Shapley Additive exPlanations (SHAP) values are introduced to assess the relationship between feature parameters and the final predicted results [37]. SHAP is a machine learning algorithm used to explain model predictions. It provides a SHAP value for each feature, indicating the contribution of that feature to the model prediction, and also shows the positive or negative impact. The advantage of SHAP values is that they can provide intuitive explanations and visualizations. The overall visualization of the feature parameters is performed, as shown in Figure 21. Each row in the figure represents a feature value and the *x*-axis represents the SHAP value. Each point represents a sample, where a redder color indicates a larger feature value and a bluer color indicates a smaller feature value. It can be intuitively observed that *K_y_* and *K_z_* are two very important features; they are basically positively correlated with the corrosion rate. That is, the higher the values of *K_y_* and *K_z_*, the higher the corrosion rate. In addition, there is a weak correlation between the two other feature parameters and the corrosion rate. The protective layer thickness is positively correlated with the corrosion rate, while the rebar diameter is negatively correlated. Furthermore, the influence of variables on the target value under the interaction of two variables is analyzed, including the SHAP values and the original values, as shown in Figure 22. It can be seen that the trend of *K_y_* and *K_z_* is monotonically increasing. Taking Figure 22a as an example for analysis, it can be seen from the figure that when *K_z_* is smaller, the color of *K_y_* is mostly blue, indicating that the value of *K_y_* is relatively small at this time. When *K_y_* is larger, the color of *K_y_* is basically red, indicating that the value of *K_y_* is increasing. Therefore, it can be inferred that the interaction coefficient between *K_y_* and *K_z_* should be a positive value. Then, the influence of each feature on the predicted value is randomly checked for one of the samples, as shown in Figure 23. Red indicates that the contribution of the feature is positive, while blue indicates that the contribution of the feature is negative. In the figure, the color bar of the rebar diameter *D* is the shortest, indicating that this parameter contributes the least to the prediction of corrosion. The color bar of the parameter *K_y_* is the longest, indicating that this parameter contributes the most to the prediction of the corrosion.

**Figure 24:**
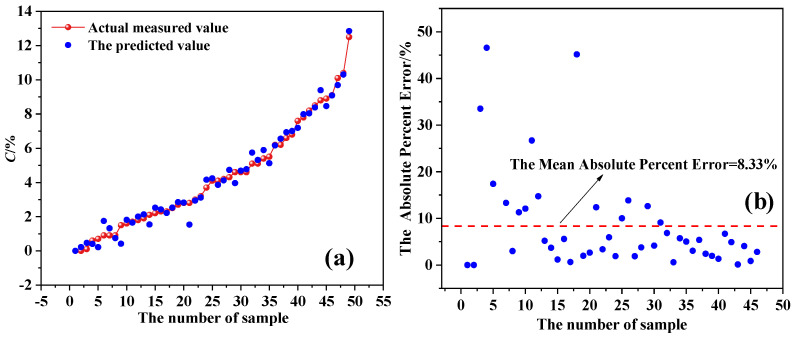
The test results. (**a**) Predicted results and (**b**) absolute percentage error of the predicted results.

The established XGBoost model is used to predict the corrosion rate *C* of 48 test samples and the prediction results are shown in Figure 24. From the figure, it can be seen that the predicted results of the XGBoost model are close to the actual values. The mean squared error (MSE) of the test set is 0.164, *R*^2^ is 0.982, the standard deviation (SD) is 0.263, and the root mean squared error (RMSE) is 0.405. The minimum absolute error between the predicted and actual results is 0.11% and the maximum absolute error is 46.6%. The average absolute percentage error is 8.33%, which is within 10%, indicating that the model has high accuracy.

## 4. Conclusions

Based on experimental and theoretical studies, the variation in the SMFL field of reinforced concrete structures with the corrosion of rebars was analyzed and the following conclusions can be drawn

1. A magnetic dipole model coupling with a multi-defect is established and the influence of the corrosion expansion force and corrosion degree on the SMFL signal is analyzed. The results show that the standard deviation of the magnetic field intensity caused by corrosion varied by up to 833%, while that caused by corrosion expansion force did not exceed 10%. Therefore, the influence of the corrosion expansion force on the detection of corrosion damage can be ignored;
2. Experimental results show that the trough positions of the Δ*By* curves and the intersection points of the Δ*Bz* curves obtained at different measurement heights are in good agreement with the positions of severe corrosion of the rebars. Compared to the △*Bz*, the severe corrosion area of rebars could be more accurately identified based on the change in △*By* curves;
3. The experimental results indicate that the SMFL curves of rebars in reinforced concrete change monotonically with the increase in the corrosion degree. Based on this characteristic of change, the proposed quantification parameters *K_y_* and *K_z_* have a good positive correlation with the corrosion degree of the rebars;
4. Based on the proposed magnetic parameters and the geometric parameters of the specimens, the corrosion degree of the specimens can be accurately determined according to the prediction model. The average absolute error of the prediction results is 8.33%, which is within 10%. It is a useful prediction for the corrosion degree of rebars in reinforced concrete structures.

## Figures and Tables

**Table 1:** The chemical component of HRB400.

Chemical Component (Mass Fraction)/%					
Brand	C	Si	Mn	S	P
HRB400	≤0.25	≤0.8	≤1.6	≤0.04	≤0.04

**Table 2:** The final actual corrosion rate for specimens.

Number	*I*/A	Current Density/µA·cm^−2^	Theoretical Corrosion Rate/%	Actual Corrosion Rate/%
B22-2	0.2	1136.82	7.6	5.5
A32-1	0.2	1136.82	11.9	14.9
A12-2	0.2	1136.82	2.8	2.7
A11-2	0.2	1515.76	5.0	5.5
A31-2	0.2	1515.76	13.8	10.6
A22-1	0.2	1136.82	10.1	10.7
A33-3	0.2	909.46	5.2	6.5
B32-3	0.2	1136.82	7.9	5.4
B12-1	0.2	1136.82	11.4	9.0

**Table 3:** Hyperparameter in the model.

Max Tree Depth	Learning Rate	Number of Base Learners
4	0.96	40

**Table 4:** Partial training sample regression test results.

Number	Actual Corrosion Rate %	Predicted Corrosion Rate %	Number	Actual Corrosion Rate %	Predicted Corrosion Rate %
1	8.8	8.8	21	1.5	1.5
2	13.1	13.1	22	2.4	2.4
3	4.9	4.9	23	3.7	3.7
4	6.6	6.6	24	8.2	8.2
5	3.9	3.9	25	2.5	2.5
6	0.6	0.6	26	4.5	4.5
7	9.4	9.4	27	0.8	0.8
8	8.5	8.5	28	2.1	2.1
9	4.0	4.0	29	3.9	3.9
10	3.8	3.8	30	2.7	2.7
11	11.0	11.0	31	1.6	1.6
12	2.3	2.3	32	8.8	8.8
13	9.1	9.1	33	10.0	10.0
14	2.8	2.7	34	0.6	0.6
15	2.2	2.2	35	1.7	1.7
16	3.2	3.2	36	4.2	4.8
17	6.3	6.3	37	4.7	4.7
18	4.1	4.1	38	0.3	0.3
19	6.8	6.8	39	4.4	4.4
20	3.9	3.9	40	5.1	5.2

## Data Availability

The data presented in this study are available from the first and corresponding author upon request. The data are not publicly available due to the policy of the data provider.
